# Latest evidence on transfer efficiency and patient outcomes in mothership versus drip-and-ship models for acute stroke management: a systematic review and meta-analysis

**DOI:** 10.3389/fneur.2026.1941406

**Published:** 2026-08-24

**Authors:** Fei Zhang, Chunlan Tian, Jian Wu, Qiang Zhang, Qiang Li

**Affiliations:** 1 Clinical Medical College, Qinghai University, Xining, China; 2 Lvliang Maternal and Child Health and Family Planning Service Center, Lvliang Maternal and Child Health Hospital, Lvliang, Shanxi, China; 3 Department of Neurosurgery, Qinghai Provincial People's Hospital, Xining, China; 4 Department of Cerebrovascular Diseases, Henan Provincial People's Hospital, Henan Provincial People's Clinical Medical School of Zhengzhou University, Zhengzhou, China

**Keywords:** drip-and-ship, endovascular thrombectomy, mothership, stroke, systematic review

## Abstract

**Background:**

Acute ischemic stroke requires urgent treatment, yet geographic disparities in specialized care necessitate two transport strategies: direct transfer to comprehensive stroke centers (mothership, MS) versus initial treatment at primary centers followed by transfer (drip-and-ship, DS). Optimal routing remains uncertain, particularly with extended treatment time windows.

**Methods:**

Systematic review and meta-analysis adhering to PRISMA/MOOSE guidelines. Literature search of PubMed, Embase, and Cochrane through October 17, 2025, identified comparative studies evaluating MS versus DS in acute stroke. Outcomes included 90-day functional independence (mRS 0-2), symptomatic intracranial hemorrhage (sICH), reperfusion success (TICI 2b-3), and 90 days mortality. Random-effects models pooled odds ratios.

**Results:**

Twenty-eight observational studies (10,059 patients) were included; no randomized controlled trial met the final inclusion criteria. MS demonstrated superior functional independence (OR 0.83, 95%CI 0.73–0.95, *p* = 0.008), with stronger effects in prospective studies. No mortality difference was observed (OR 1.05, 95%CI 0.92–1.21). MS significantly increased hemorrhage risk (OR 1.41, 95%CI 1.12–1.77, *p* = 0.003). Reperfusion rates did not differ between models.

**Conclusion:**

The mothership model improves functional outcomes but elevates hemorrhage risk without mortality benefit. Direct transport to comprehensive stroke centers is recommended when geographically feasible, accompanied by intensive post-procedural monitoring. Rural settings require tailored, context-specific strategies.

## Introduction

Acute ischemic stroke (AIS) is one of the most time-sensitive medical emergencies, with each minute of treatment delay resulting in the loss of approximately 1.9 million neurons (1). Endovascular thrombectomy (EVT) has completely changed the treatment landscape of large vessel occlusion (LVO) with a revascularization rate as high as 80–90% (2), which is far superior to the 25–30% achieved by intravenous thrombolysis (IVT) (3). However, the geographical distribution of comprehensive stroke center (CSC) capable of EVT is uneven, giving rise to two prehospital triage paradigms: “mothership (MS)” and “drip-and-ship (DS)” (4). The former involves transporting patients directly to CSC regardless of distance. The latter involves initial assessment and IVT at the nearest primary stroke center (PSC), followed by transfer to a CSC for possible EVT.

In recent years, advances in perfusion imaging have extended the EVT time window to 24 h, shifting the selection criteria from “time-based” to “tissue-based,” allowing some patients to benefit from intervention even 24 h after symptom onset (5–7). This has made the relative advantages of the two models highly significant: while the MS model theoretically reduces the time to treatment for LVO patients, it may increase costs and the risk of overcrowding (8); the DS model, though cost-effective and ensuring timely IVT, may compromise the best outcomes for some patients. Rural areas face even greater challenges due to differences in infrastructure (9), while innovations such as mobile stroke units (10–12), AI - assisted decision—making, and physician - to - physician transfer protocols are blurring traditional boundaries (13).

Given the complexity of these considerations and the evolving nature of stroke care delivery, this systematic review and meta-analysis aims to comprehensively evaluate and compare the existing evidence for the MS and DS models in acute stroke management. By synthesizing data from the latest studies and considering the impact of evolving treatment paradigms, this analysis will provide the evidence needed by clinicians, healthcare managers, and policymakers to optimize stroke care delivery systems and improve patient outcomes across different healthcare settings.

## Methods

This meta-analysis conforms to the Preferred Reporting Items for Systematic Reviews and Meta-Analyses (PRISMA) guidelines (14) and was written in accordance with the Meta-analysis of Observational Studies in Epidemiology (MOOSE) guidelines (15). The study has been registered with PROSPERO, with the registration number: CRD420251167458.

### Data sources and literature searches

We conducted electronic literature searches in PubMed, Embase, and the Cochrane Library from inception to October 17, 2025. The search terms included “stroke,” “thrombectomy,” “Mothership,” “Drip and Ship,” and other related terms. In addition, we manually reviewed the reference lists of the identified publications for additional studies. The detailed search strategy is provided in the Supplementary material.

### Study selection

Eligible study designs included observational cohort studies and randomized controlled trials (RCTs) that directly compared the two transport models in patients with AIS. Eligible studies were required to report at least one of the following outcomes: (1) modified Rankin Scale (mRS) score of 0-2 at 90 days; (2) symptomatic intracranial hemorrhage (sICH); (3) Thrombolysis in Cerebral Infarction (TICI) score of 2b-3; or (4) 90-day mortality. Only full-text articles published in peer-reviewed journals were included. Conference abstracts, case reports, letters, animal experiments, and reviews not relevant to the topic were excluded.

### Data extraction and quality assessment

Two researchers independently performed data extraction and quality assessment for the included studies. Disagreements were resolved through discussion with the corresponding author. The extracted data included: author, year, study design, study duration, study region, number of patients included, study group, mRS 0-2 at 90 days, TICI 2b-3, sICH, and 90-day mortality. We used the Newcastle-Ottawa Scale (NOS) to evaluate the quality of included studies, and studies with a score of 7 or higher were defined as high-quality studies. If at least 10 studies were available for analysis, we used funnel plots to assess publication bias.

### Data synthesis and analysis

All extracted data were imported into Review Manager (version 5.4.1) for pooled analysis. Odds ratios (ORs) together with their 95% confidence intervals (CI) were estimated under a random-effects model for every outcome. Between-study heterogeneity was quantified with the *I*^2^ statistic and tested by the Cochrane *Q* test; *I*^2^ < 25% indicated low heterogeneity, whereas *I*^2^ ≥ 50% denoted substantial heterogeneity. Publication bias was visually inspected and statistically tested with funnel-plot–based methods; a two-sided *p* value < 0.05 was considered statistically significant.

To bolster the credibility of our findings, we conducted pre-specified subgroup and sensitivity analyses. In the subgroup analysis, studies were stratified by study design: prospective cohorts versus retrospective cohorts. We hypothesized that prospective studies, with their standardized protocols and prospective data collection, would yield more precise estimates of the mothership effect, whereas retrospective studies might introduce selection or information bias, potentially inflating or diluting the observed benefit. For outcomes showing substantial heterogeneity, we performed a leave-one-out sensitivity analysis by sequentially excluding individual studies and recalculating the pooled effect estimate and *I*^2^ statistic. This analysis was intended to evaluate whether the pooled result and statistical heterogeneity were disproportionately influenced by individual studies, rather than to identify or eliminate specific clinical sources of heterogeneity.

## Results

**Figure 1:**
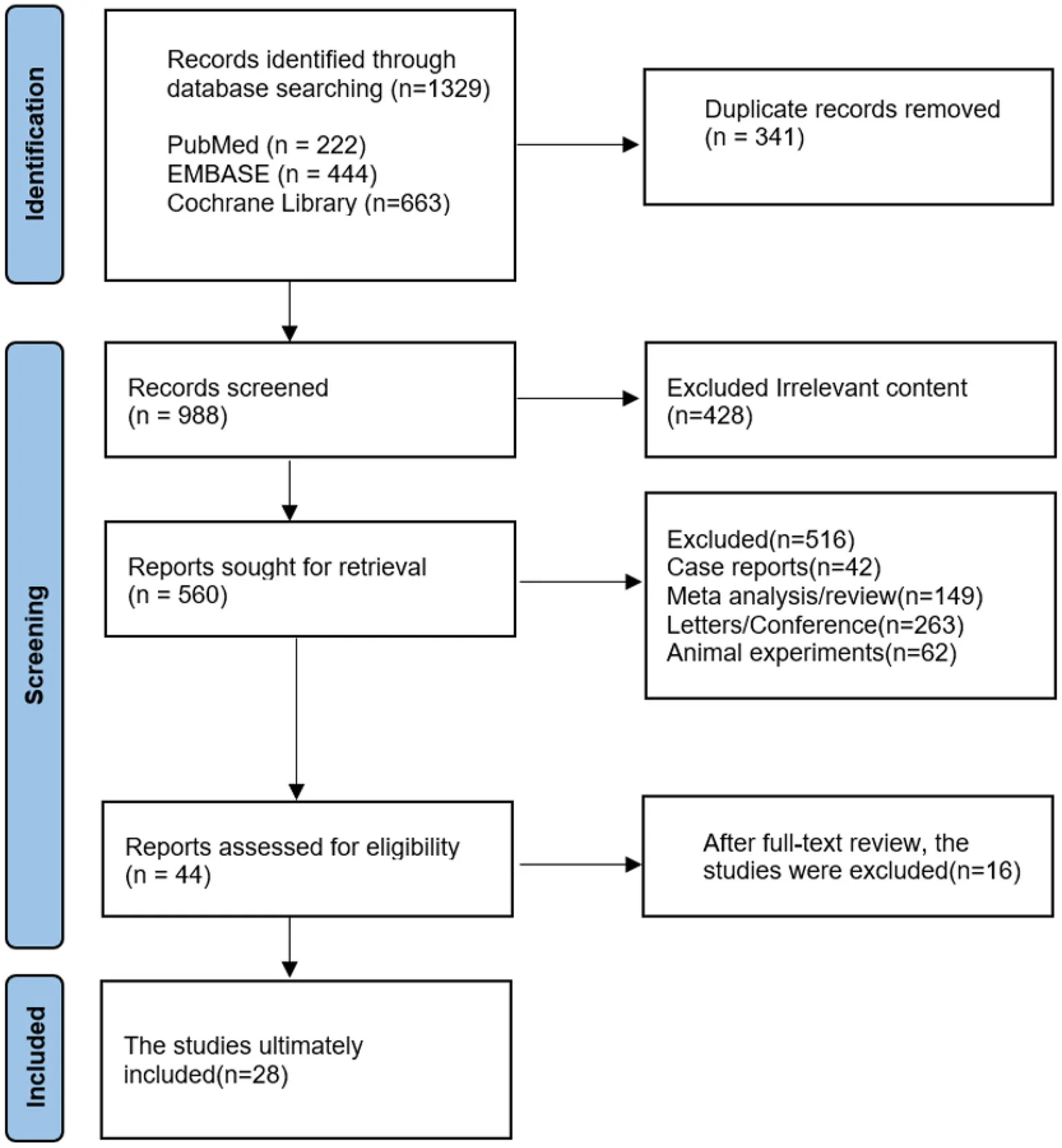
PRISMA flowchart illustrating the systematic review process of study selection.

A total of 1,329 records were initially retrieved from three databases. After removing 341 duplicates, 988 articles remained for further screening. Subsequently, 944 studies were excluded on the basis of title and abstract, and 28 publications ultimately met the inclusion criteria following full-text review (16–43). The detailed screening process is illustrated in Figure 1.

### Study characteristics and quality assessment

Among the 28 studies included, none was a randomized controlled trial. All included studies were observational: 21 were retrospective (16–20, 22, 23, 25–36, 39, 40), six were prospective (24, 37, 38, 41–43), and one included both retrospective and prospective cohorts (21). Collectively, 10 059 stroke patients were enrolled: 4 361 in the DS group and 5 698 in the MS group. The essential characteristics of each study are summarized in Supplementary Material Table 1. Study quality was assessed with the NOS; studies scoring ≥7 were considered high-quality. Twenty-one studies met this criterion, whereas seven scored 6 points (16, 19, 30, 32, 33, 39, 41). Notably, two studies achieved the maximum score of 9 (31, 37). Detailed scoring is provided in Supplementary Material Table 2. These findings indicate that the present meta-analysis is based on evidence of satisfactory reliability.

The geographical context of the included studies was also reviewed because transport distance and regional stroke-system organization may modify the relative effects of MS and DS. Only a limited number of studies explicitly characterized their study population as rural or urban. For example, Raquin et al. (38) evaluated the two transport strategies in a rural setting, whereas Ferreira Cristina et al. (22) investigated patients within an urban region. For most studies, a formal rural/urban classification was not explicitly reported. We therefore did not assign geographical categories retrospectively when these were not specified by the original investigators, and the available data were insufficient for a formal rural-versus-urban subgroup meta-analysis.

### Outcome characteristics

**Figure 2:**
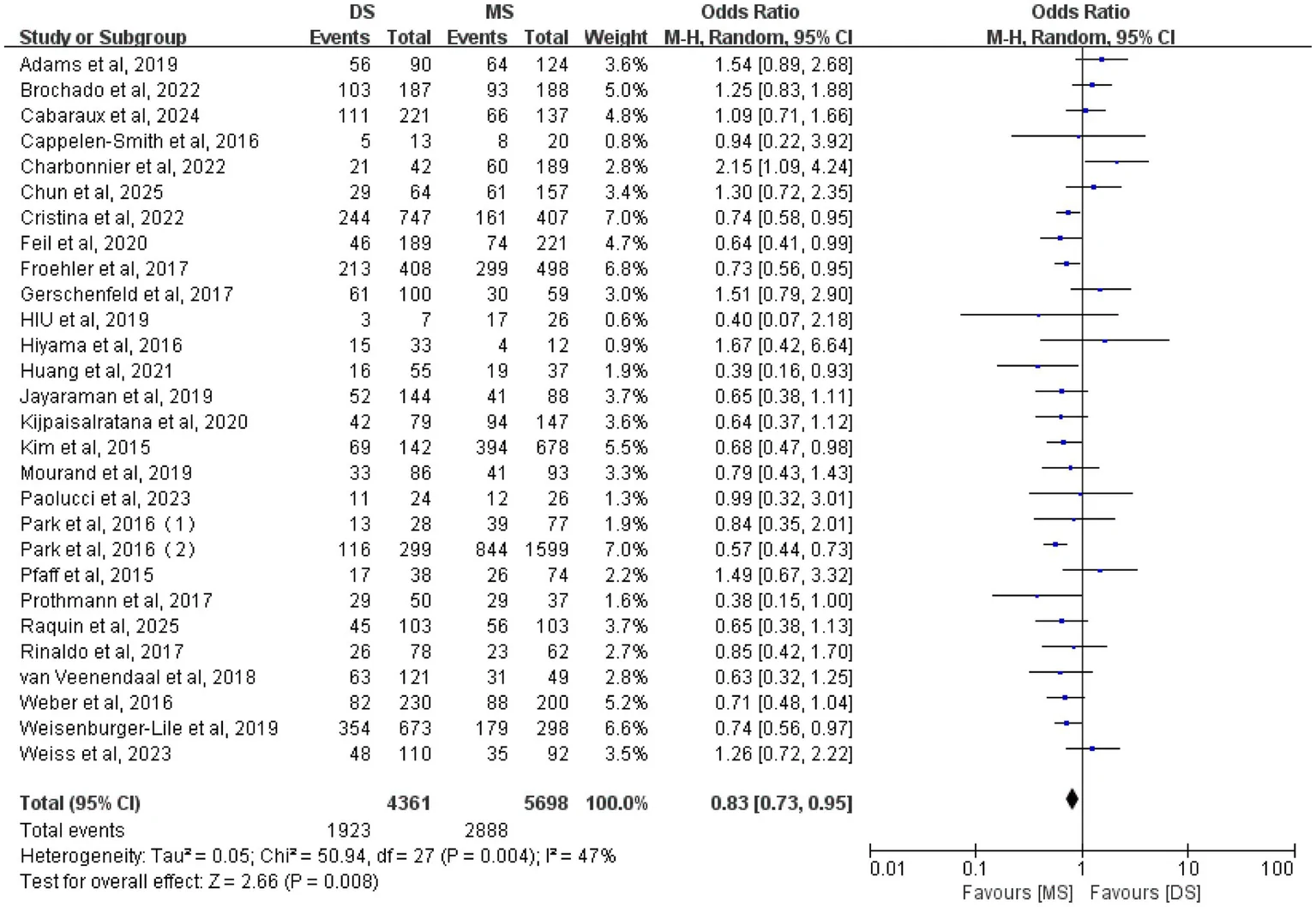
Forest plot comparing odds ratios of 90-day functional independence (mRS 0–2) between drip-and-ship (DS) and mothership (MS) models across 28 studies.

Across all 28 included studies, the proportion of patients achieving a 90-day mRS score of 0–2—an established indicator of functional independence after stroke—was reported. Pooled analysis with a random-effects model showed that the MS group attained significantly better functional independence than the DS group (OR = 0.83, 95% CI 0.73–0.95, *p* = 0.008, *I*^2^ = 47%; Figure 2). Moderate heterogeneity suggests that inter-study variability may have influenced this estimate.

**Figure 3:**
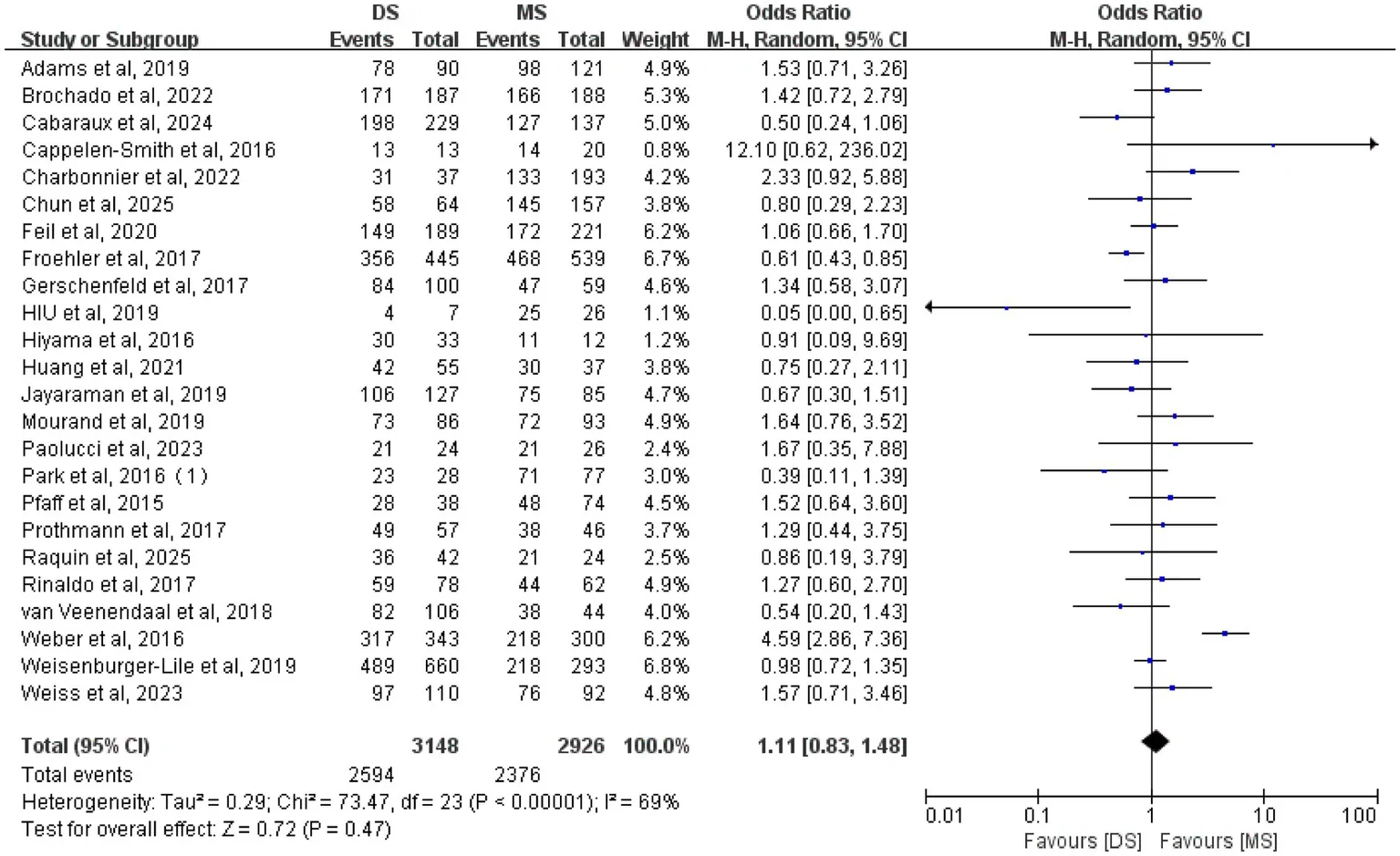
Forest plot comparing odds ratios of successful reperfusion (TICI 2b–3) between DS and MS models across 24 studies.

**Figure 4:**
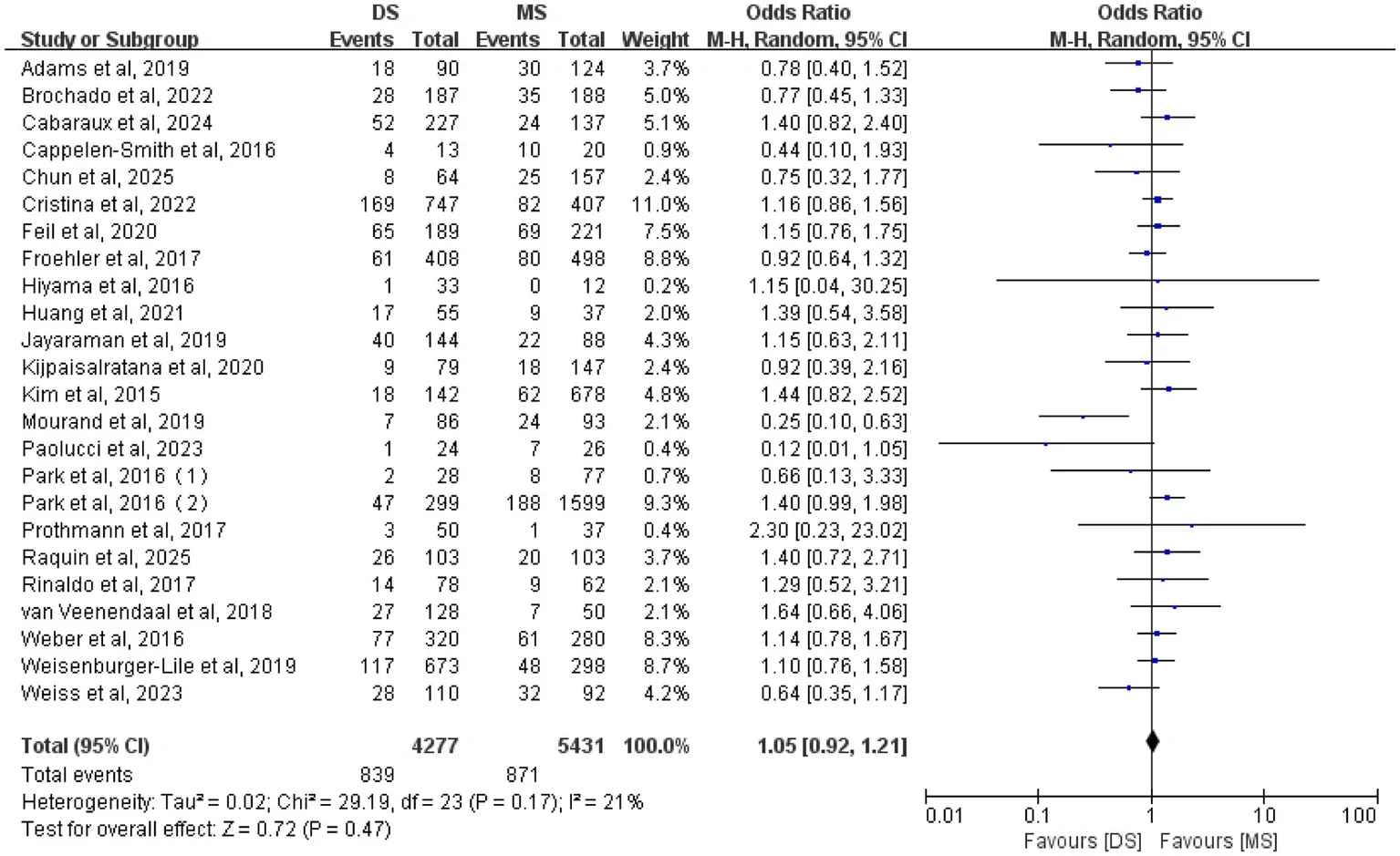
Forest plot comparing odds ratios of 90-day mortality between DS and MS models across 24 studies.

**Figure 5:**
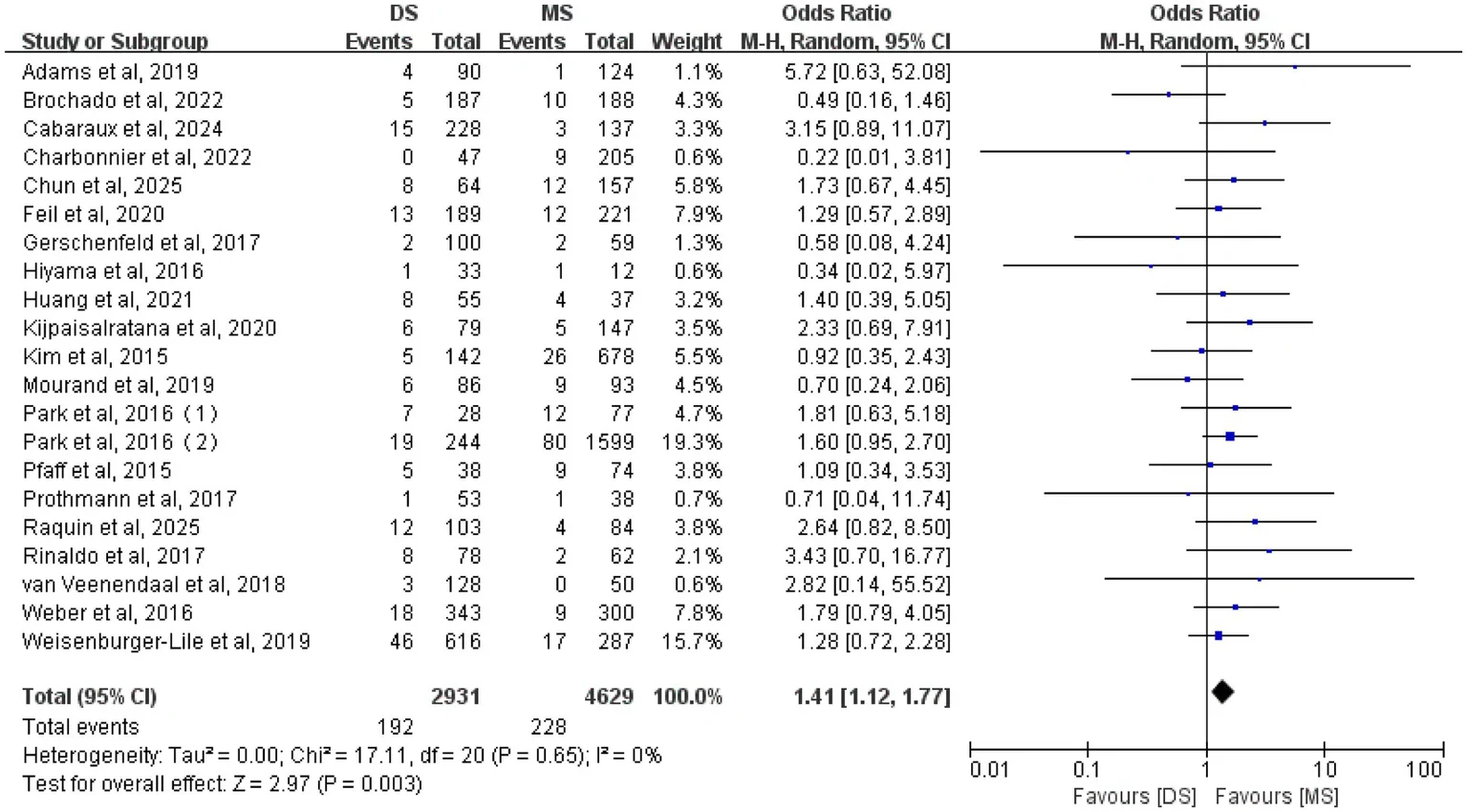
Forest plot comparing odds ratios of symptomatic intracranial hemorrhage (sICH) between DS and MS models across 21 studies.

Twenty-four studies provided data on successful vascular reperfusion. No significant between-group difference was observed (OR = 1.11, 95% CI 0.83–1.48, *p* = 0.47, *I*^2^ = 69%; Figure 3). The substantial heterogeneity indicates appreciable between-study variability and may reflect clinical and methodological differences, including reperfusion definitions, assessment timing, patient selection, and interventional protocols. Twenty-four studies reported 90-day mortality. Meta-analysis demonstrated no significant difference in the risk of death between the MS and DS groups (OR = 1.05, 95% CI 0.92–1.21, *p* = 0.47, *I*^2^ = 21%; Figure 4). The low heterogeneity indicates good inter-study consistency and lends greater credibility to this finding. With respect to safety, the MS group exhibited a significantly higher incidence of sICH. Data pooled from 21 studies revealed a 41% relative increase in sICH risk in the MS group compared with the DS group (OR = 1.41, 95% CI 1.12–1.77, *p* = 0.003, *I*^2^ = 0%; Figure 5). The absence of detectable heterogeneity underscores high consistency across studies and strengthens the reliability of this observation. To evaluate potential publication bias, funnel plots were constructed for all primary outcomes (Supplementary Figures S1–S4, Supplementary Material). Overall, no overt asymmetry suggestive of publication bias was identified; nevertheless, the limited number of studies for some outcomes precludes definitive exclusion of small-study effects.

In summary, the MS strategy conferred a significant advantage in functional independence at 90 days relative to DS, yet it did not improve rates of successful reperfusion or reduce 90-day mortality, and it was associated with a markedly higher risk of sICH. Therefore, clinicians should carefully weigh individual patient risks and benefits when selecting a therapeutic approach, and they should implement intensive post-procedural monitoring to minimize hemorrhagic complications.

### Subgroup analysis results

We examined whether study design (prospective vs. retrospective) influenced the pooled effect of the MS mode compared to the DS mode in terms of good functional outcomes. Among the 27 cohorts included, 21 were retrospective studies (7,036 patients, 3,264 events), and 6 were prospective studies (2,808 patients, 1,457 events). Supplementary Figure S5 shows that in both subgroups, the effect direction consistently favored the “MS” mode; however, the magnitude and precision of the effect differed. In retrospective studies, the pooled OR was 0.86 (95% CI 0.72–1.03, *p* = 0.10), with moderate heterogeneity (*I*^2^ = 52%). In prospective studies, the pooled OR was 0.74 (95% CI 0.62–0.87, *p* = 0.0004), with lower heterogeneity (*I*^2^ = 10%). Although the subgroup difference test was not statistically significant (*p* = 0.23), the effect estimate in prospective cohort studies was more precise, with a narrower confidence interval and negligible between-study variance, supporting higher reliability of the effect estimate in such designs.

### Sensitivity analysis

Substantial heterogeneity was observed in the pooled analysis of successful reperfusion (*I*^2^ = 69%). We therefore performed a leave-one-out sensitivity analysis to evaluate the influence of individual studies on the pooled estimate and between-study heterogeneity. The exclusion of Weber et al. (41), which exerted a disproportionate influence on heterogeneity, reduced *I*^2^ from 69 to 37%. After exclusion of this study, the pooled effect remained non-significant (23 studies; OR = 1.01, 95% CI 0.81–1.25, *p* = 0.96; Supplementary Figure S6). Thus, the overall conclusion of no significant difference in successful reperfusion between MS and DS was robust to the exclusion of this influential study. Importantly, this sensitivity analysis does not eliminate potential clinical heterogeneity related to differences in reperfusion definitions, assessment timing, patient selection, or interventional protocols across the included studies.

## Discussion

### Strengths of this study

This systematic review and meta-analysis provides a comprehensive evaluation comparing mothership and drip-and-ship models for acute ischemic stroke management. Our analysis included 28 observational studies with over 10,000 patients. The inclusion of both retrospective and prospective observational cohorts, along with rigorous quality assessment using the Newcastle-Ottawa Scale, strengthens the reliability of our findings. The most significant contribution of this work is the demonstration that the mothership model consistently provides better functional outcomes (OR = 0.83, 95% CI 0.73–0.95, *p* = 0.008), while also revealing the nuanced trade-offs between faster treatment access and increased hemorrhagic complications. Our subgroup analysis further clarified that prospective studies showed stronger effects with lower heterogeneity, suggesting that well-designed prospective research may provide more precise estimates of the mothership advantage.

### Results in relation to other studies and reviews

Our findings align remarkably well with previous systematic reviews examining this topic. The 2021 meta-analysis by Mohamed and colleagues, which analyzed 13 studies with 7,824 patients, also found that mothership was superior to drip-and-ship for functional independence, with similar odds ratios for good outcomes (OR = 1.34 in their analysis versus our OR = 0.83, noting the inverse relationship due to different reference groups) (44). Similarly, the 2020 meta-analysis by Romoli and colleagues, encompassing 18 studies with 7,017 patients, demonstrated consistent superiority of the mothership model for functional independence (OR = 1.34, 95% CI 1.16–1.55) (45). Both previous reviews also found no significant differences in mortality rates between the two models, which matches our findings exactly. The consistency across three major meta-analyses spanning different time periods and including partially overlapping study populations strengthens the evidence base for preferential mothership routing when geographically feasible.

However, our study reveals important differences from previous work that warrant careful consideration. Unlike the earlier reviews, we found a significantly higher risk of symptomatic intracranial hemorrhage in the mothership group (OR = 1.41, 95% CI 1.12–1.77, *p* = 0.003), a finding that was not prominent in the 2020 and 2021 meta-analyses. This discrepancy may reflect the inclusion of more recent studies with better hemorrhage detection protocols and standardized reporting criteria. Additionally, recent studies by Raquin et al. (38) and Weiss et al. (43) suggest that in rural settings, the differences between models may be less pronounced, with some showing comparable outcomes between paradigms. The emergence of hybrid models such as “drive-the-doctor” and mobile stroke units has also complicated the traditional binary comparison, with simulation studies suggesting these approaches may bridge the gap between models (46).

The divergent findings regarding hemorrhagic complications likely stem from several factors. First, the extended time window for thrombectomy (up to 24 h in modern practice) means that mothership patients may present with more established infarcts and blood–brain barrier disruption, increasing hemorrhage risk upon reperfusion. Second, the concentration of high-acuity patients at comprehensive stroke centers may lead to more aggressive treatment protocols and closer monitoring, resulting in higher detection rates of hemorrhagic complications. Third, the evolution of imaging technology and standardized hemorrhage classification systems has improved detection sensitivity over time. The rural–urban differences observed in recent studies probably reflect geographical realities—in remote areas, the time penalty for direct transport to comprehensive centers may offset the benefits of avoiding interhospital transfer, particularly when primary stroke centers have well-established rapid transfer protocols. These regional variations highlight the importance of context-specific stroke system design rather than universal application of a single model.

Our findings have important implications for clinical practice and future research. The consistent functional outcome advantage of mothership routing supports direct transport to comprehensive stroke centers when travel times are reasonable. However, the increased hemorrhage risk requires heightened vigilance and standardized post-procedure monitoring protocols. For rural and remote areas, the development of thrombectomy-capable stroke centers and mobile interventional teams may provide the optimal balance between timely treatment and system efficiency. Future research should focus on identifying patient subgroups who might benefit most from each approach, developing predictive models for optimal routing decisions, and evaluating the cost-effectiveness of different stroke system configurations. Randomized controlled trials comparing these models in specific geographical contexts would provide the highest level of evidence, though logistical challenges remain substantial. The integration of artificial intelligence and machine learning algorithms for transfer decision support represents a particularly promising avenue for optimizing stroke care delivery in the coming decade.

### Limitations

First, the predominance of retrospective cohorts (75% of included studies) introduces potential selection bias and confounding by indication, as patient routing was not randomized; no eligible randomized controlled trial was included in the final meta-analysis. Second, substantial heterogeneity in interventional protocols, device technologies, and outcome definitions across studies may limit comparability. Third, geographic variability restricts generalizability, particularly to rural and remote settings where prolonged transport distances may offset the MS advantage. Moreover, most included studies did not explicitly classify their study populations according to rural versus urban setting, precluding a reliable geographical subgroup meta-analysis; therefore, the applicability of the pooled estimates to different regional stroke-care systems should be interpreted cautiously. Fourth, although funnel plots showed no overt asymmetry, the limited number of studies for some outcomes precludes definitive exclusion of publication bias. Fifth, insufficient data on cost-effectiveness, healthcare resource utilization, and long-term outcomes hinder comprehensive health economic evaluation. Finally, the rapid evolution of stroke care—including mobile stroke units and extended treatment windows—means some studies reflect outdated practice paradigms.

## Conclusion

The mothership model improves 90-day functional independence versus drip-and-ship for acute ischemic stroke but increases symptomatic intracranial hemorrhage risk without mortality benefit. This supports direct transport to comprehensive stroke centers when geographically feasible, with vigilant post-procedural monitoring. However, rural areas face transfer limitations; future research should prioritize physician-transfer models and context-specific strategies to optimize stroke care delivery.
