# Supplementary material for: Latest evidence on transfer efficiency and patient outcomes in mothership versus drip-and-ship models for acute stroke management: a systematic review and meta-analysis
Source: Front Neurol. 2026 Aug 24;17:1941406. doi: 10.3389/fneur.2026.1941406 (PMC13550722; doi:10.3389/fneur.2026.1941406)
Supplement: Supplementary file 1 [file Data_Sheet_1.docx]

**Supplemental Materials**

**Search strategy**

**Pubmed**

("stroke"[MeSH Terms] OR "stroke*"[Title/Abstract] OR "ischemia*"[Title/Abstract] OR "ischaemia*"[Title/Abstract] OR "infarct*"[Title/Abstract] OR "cerebrovascular*"[Title/Abstract] OR "vessel occlusion"[Title/Abstract] OR "Apoplexy"[Title/Abstract]) AND ("thrombectomy"[MeSH Terms] OR "thrombectomy*"[Title/Abstract] OR "endovascular procedures"[Title/Abstract] OR "Endovascular"[Title/Abstract] OR "Intravascular"[Title/Abstract] OR "embolectomy*"[Title/Abstract] OR "thrombolysis*"[Title/Abstract] OR "mechanical clot disruption*"[Title/Abstract]) AND ("Mothership"[Title/Abstract] OR ("Drip"[Title/Abstract] AND "Ship"[Title/Abstract]) OR ("MD"[Title/Abstract] AND "DS"[Title/Abstract]) OR "primary center"[Title/Abstract] OR "organization model"[Title/Abstract])

**Embase**

#1 'cerebrovascular accident'/exp

#2 stroke:ab,ti OR stroke*:ab,ti OR ischemia*:ab,ti OR ischaemia*:ab,ti OR infarct*:ab,ti OR cerebrovascular*:ab,ti OR 'vessel occlusion':ab,ti OR apoplexy:ab,ti

#3 #1 OR #2

#4 'thrombectomy'/exp

#5 thrombectomy*:ab,ti OR 'endovascular procedures':ab,ti OR endovascular:ab,ti OR intravascular:ab,ti OR embolectomy*:ab,ti OR thrombolysis*:ab,ti OR 'mechanical clot disruption*':ab,ti

#6 #4 OR #5

#7 mothership:ab,ti OR (drip:ab,ti AND ship:ab,ti) OR (md:ab,ti AND ds:ab,ti) OR 'primary center':ab,ti OR 'organization model':ab,ti

#8 #3 AND #6 AND #7

**Cochrane**

#1 MeSH descriptor: [Stroke] explode all trees

#2 (stroke*):ti,ab,kw OR (ischemia*):ti,ab,kw OR (ischaemia*):ti,ab,kw OR (infarct*):ti,ab,kw OR (cerebrovascular*):ti,ab,kw

#3 (vessel occlusion):ti,ab,kw OR (Apoplexy):ti,ab,kw

#4 #1 OR #2 OR #3

#5 MeSH descriptor: [Thrombectomy] explode all trees

#6 (thrombectomy*):ti,ab,kw OR (endovascular procedures):ti,ab,kw OR (Endovascular):ti,ab,kw OR (Intravascular):ti,ab,kw OR (embolectomy*):ti,ab,kw

#7 (thrombolysis*):ti,ab,kw OR (mechanical clot disruption*):ti,ab,kw

#8 #5 OR #6 OR #7

#9 (Mothership):ti,ab,kw OR (Drip and Ship):ti,ab,kw OR (MD and DS):ti,ab,kw OR (primary center):ti,ab,kw OR (organization model):ti,ab,kw

#10 #4 AND #8 AND #9

**Table 1. Summary of the Data Extracted From the Studies**

| Author, year | Study design | Duration of the study | Study region | Patients | Study Group | mRS 0-2 at 90 days | TICI 2b-3 | sICH | mortality at 90 days |
| --- | --- | --- | --- | --- | --- | --- | --- | --- | --- |
| Adams et al, 2019 | Retrospective | 2014.01-2017.12 | Ireland | 214 | MS: 124 | 64/124 | 98/121 | 1/124 | 30/124 |
|  |  |  |  |  | DS: 90 | 56/90 | 78/90 | 4/90 | 18/90 |
| Brochado et al, 2022 | Retrospective | 2018.01-2020.12 | Spain | 375 | MS: 188 | 93/188 | 166/188 | 10/188 | 35/188 |
|  |  |  |  |  | DS: 187 | 103/187 | 171/187 | 5/187 | 28/187 |
| Cabaraux et al,2024 | Retrospective | 2017.01-2022.12 | Belgium | 366 | MS: 137 | 66/137 | 127/137 | 3/137 | 24/137 |
|  |  |  |  |  | DS: 229 | 111/221 | 198/229 | 15/228 | 52/227 |
| Cappelen-Smith et al, 2016 | Retrospective | 2014.01-2015.12 | Australia | 33 | MS: 20 | 8/20 | 14/20 | NP | 10/20 |
|  |  |  |  |  | DS: 13 | 5/13 | 13/13 | NP | 4/13 |
| Charbonnier et al, 2022 | Retrospective | 2015.01-2018.12 | France | 252 | MS: 205 | 60/189 | 133/193 | 9/205 | NP |
|  |  |  |  |  | DS: 47 | 21/42 | 31/37 | 0/47 | NP |

**Table 1. Continued**

| Chun et al, 2025 | Retrospective,  Prospective | 2019.01-2024.06 | Taiwan | 1063 | MS: 157 | 61/157 | 145/157 | 12/157 | 25/157 |
| --- | --- | --- | --- | --- | --- | --- | --- | --- | --- |
|  |  |  |  |  | DS: 64 | 29/64 | 58/64 | 8/64 | 8/64 |
| Cristina et al, 2022 | Retrospective | 2018.01-2020.12 | Portugal | 1154 | MS: 747 | 161/407 | NP | NP | 82/407 |
|  |  |  |  |  | DS: 407 | 244/747 | NP | NP | 169/747 |
| Feil et al, 2020 | Retrospective | 2015.01-2018.05 | Germany | 410 | MS: 221 | 74/221 | 172/221 | 12/221 | 69/221 |
|  |  |  |  |  | DS: 189 | 46/189 | 149/189 | 13/189 | 65/189 |
| Froehler et al, 2017 | Prospective | 2014.08-2016.06 | USA | 984 | MS: 539 | 299/498 | 468/539 | NP | 80/498 |
|  |  |  |  |  | DS: 445 | 213/408 | 156/445 | NP | 61/408 |
| Gerschenfeld et al, 2017 | Retrospective | 2013.01-2016.04 | France | 159 | MS: 59 | 30/59 | 47/59 | 2/59 | NP |
|  |  |  |  |  | DS: 100 | 61/100 | 84/100 | 2/100 | NP |
| HIU et al, 2019 | Retrospective | 2010-2017 | Japan | 33 | MS: 26 | 17/26 | 25/26 | NP | NP |
|  |  |  |  |  | DS: 7 | 3/7 | 4/7 | NP | NP |

**Table 1. Continued**

| Hiyama et al, 2016 | Retrospective | 2013.09-2015.08 | Japan | 45 | MS: 12 | 4/12 | 11/12 | 1/12 | 0/12 |
| --- | --- | --- | --- | --- | --- | --- | --- | --- | --- |
|  |  |  |  |  | DS: 33 | 15/33 | 30/33 | 1/33 | 1/33 |
| Huang et al, 2021 | Retrospective | 2017.04-2018.07 | China | 92 | MS: 37 | 19/37 | 30/37 | 4/37 | 9/37 |
|  |  |  |  |  | DS: 55 | 16/55 | 42/55 | 8/55 | 17/5537 |
| Jayaraman et al, 2019 | Retrospective | 2016.01-2017.12 | USA | 232 | MS: 88 | 41/88 | 75/85 | NP | 22/88 |
|  |  |  |  |  | DS: 144 | 52/144 | 106/127 | NP | 40/144 |
| Kijpaisalratana et al, 2020 | Retrospective | 2016.01-2017.12 | Thailand | 226 | MS: 147 | 94/147 | NP | 5/147 | 18/147 |
|  |  |  |  |  | DS: 79 | 42/79 | NP | 6/79 | 9/79 |
| Kim et al, 2015 | Retrospective | 2011.01-2012.12 | Korea | 820 | MS: 678 | 394/678 | NP | 26/678 | 62/678 |
|  |  |  |  |  | DS: 142 | 69/142 | NP | 5/142 | 12/142 |
| Mourand et al, 2019 | Retrospective | 2010.09-2012.12 | France | 179 | MS: 93 | 41/93 | 72/93 | 9/93 | 24/93 |
|  |  |  |  |  | DS: 86 | 33/86 | 73/86 | 6/86 | 7/86 |

**Table 1. Continued**

| Paolucci et al, 2023 | Retrospective | 2019-2020 | Italy | 50 | MS: 26 | 12/26 | 21/26 | NP | 7/26 |
| --- | --- | --- | --- | --- | --- | --- | --- | --- | --- |
|  |  |  |  |  | DS: 24 | 11/24 | 21/24 | NP | 1/24 |
| Park et al, 2016（1） | Retrospective | 2011.01-2014.04 | Korea | 105 | MS: 77 | 39/77 | 71/77 | 12/77 | 8/77 |
|  |  |  |  |  | DS: 28 | 13/28 | 23/28 | 7/28 | 2/28 |
| Park et al, 2016（2） | Retrospective | 2011.01-2014.03 | Korea | 1898 | MS: 1599 | 844/1599 | NP | 80/1599 | 188/1599 |
|  |  |  |  |  | DS: 299 | 116/299 | NP | 19/244 | 47/299 |
| Pfaff et al, 2015 | Retrospective | 2010.06-2013.12 | Germany | 112 | MS: 74 | 26/74 | 48/74 | 9/74 | NP |
|  |  |  |  |  | DS: 38 | 17/38 | 28/38 | 5/38 | NP |
| Prothmann et al, 2017 | Prospective | 2013.02-2015.02 | Germany | 109 | MS: 46 | 29/37 | 38/46 | 1/38 | 1/37 |
|  |  |  |  |  | DS: 57 | 29/50 | 49/57 | 1/53 | 3/50 |
| Raquin et al, 2025 | Prospective | 2019.09-2021.03 | France | 206 | MS: 103 | 56/103 | 21/24 | 4/84 | 20/103 |
|  |  |  |  |  | DS: 103 | 45/103 | 36/42 | 12/103 | 26/103 |

**Table 1. Continued**

| Rinaldo et al, 2017 | Retrospective | 2012.10-2016.06 | USA | 140 | MS: 62 | 23/62 | 44/62 | 2/62 | 9/62 |
| --- | --- | --- | --- | --- | --- | --- | --- | --- | --- |
|  |  |  |  |  | DS: 78 | 26/78 | 59/78 | 8/78 | 14/78 |
| van Veenendaal et al, 2018 | Retrospective | 2015.09-2016.09 | Australia | 178 | MS: 50 | 31/49 | 38/44 | 0/50 | 7/50 |
|  |  |  |  |  | DS: 128 | 63/121 | 80/106 | 3/128 | 27/128 |
| Weber et al, 2016 | Prospective | 2012.06-2013.08 | Germany | 643 | MS: 300 | 88/200 | 218/300 | 9/300 | 61/280 |
|  |  |  |  |  | DS: 343 | 82/230 | 317/343 | 18/343 | 77/320 |
| Weisenburger-Lile et al, 2019 | Prospective | 2012.12-2016.12 | France | 971 | MS: 298 | 179/298 | 218/293 | 17/287 | 48/298 |
|  |  |  |  |  | DS: 673 | 354/673 | 489/660 | 46/616 | 117/673 |
| Weiss et al, 2023 | Prospective | 2016.06-2018.05 | Germany | 202 | MS: 92 | 35/92 | 76/92 | NP | 32/92 |
|  |  |  |  |  | DS: 110 | 48/110 | 97/110 | NP | 28/110 |

mRS: modified Rankin scale, TICI: Thrombolysis in Cerebral Infarction Scale, sICH: symptomatic intracranial hemorrhage, MS: mothership, DS: drip-to-ship, NP: no reported.

**Table 2. Quality assessment of included studies with the** **Newcastle–Ottawa Scale.**

| Author, year | Selection | Comparability | Outcome | Overall score |
| --- | --- | --- | --- | --- |
| Adams et al, 2019 | ★★★ | ★ | ★★ | ★★★★★★ |
| Brochado et al, 2022 | ★★★★ | ★★ | ★★ | ★★★★★★★★ |
| Cabaraux et al,2024 | ★★★★ | ★ | ★★ | ★★★★★★★ |
| Cappelen-Smith et al, 2016 | ★★★ | ★ | ★★ | ★★★★★★ |
| Charbonnier et al, 2022 | ★★★★ | ★ | ★★ | ★★★★★★★ |
| Chun et al, 2025 | ★★★★ | ★★ | ★★ | ★★★★★★★★ |
| Cristina et al, 2022 | ★★★★ | ★★ | ★★ | ★★★★★★★★ |
| Feil et al, 2020 | ★★★★ | ★★ | ★★ | ★★★★★★★★ |
| Froehler et al, 2017 | ★★★★ | ★★ | ★★ | ★★★★★★★★ |
| Gerschenfeld et al, 2017 | ★★★ | ★★ | ★★★ | ★★★★★★★★ |
| HIU et al, 2019 | ★★★ | ★★ | ★★★ | ★★★★★★★★ |
| Hiyama et al, 2016 | ★★★ | ★★ | ★★★ | ★★★★★★★★ |
| Huang et al, 2021 | ★★★ | ★★ | ★★★ | ★★★★★★★★ |
| Jayaraman et al, 2019 | ★★★ | ★★ | ★★★ | ★★★★★★★★ |
| Kijpaisalratana et al, 2020 | ★★★ | ★ | ★★ | ★★★★★★ |
| Kim et al, 2015 | ★★★★ | ★★ | ★★★ | ★★★★★★★★★ |
| Mourand et al, 2019 | ★★★ | ★ | ★★ | ★★★★★★ |
| Paolucci et al, 2023 | ★★★ | ★ | ★★ | ★★★★★★ |
| Park et al, 2016（1） | ★★★★ | ★★ | ★★ | ★★★★★★★★ |
| Park et al, 2016（2） | ★★★★ | ★★ | ★ | ★★★★★★★ |
| Pfaff et al, 2015 | ★★★★ | ★★ | ★★ | ★★★★★★★★ |
| Prothmann et al, 2017 | ★★★★ | ★★ | ★★★ | ★★★★★★★★★ |
| Raquin et al, 2025 | ★★★★ | ★★ | ★ | ★★★★★★★ |

**Table 2. Continued**

| Rinaldo et al, 2017 | ★★★ | ★★ | ★ | ★★★★★★ |
| --- | --- | --- | --- | --- |
| van Veenendaal et al, 2018 | ★★★★ | ★★ | ★★ | ★★★★★★★★ |
| Weber et al, 2016 | ★★★★ | / | ★★ | ★★★★★★ |
| Weisenburger-Lile et al, 2019 | ★★★★ | ★★ | ★ | ★★★★★★★ |
| Weiss et al, 2023 | ★★★★ | ★★ | ★★ | ★★★★★★★★ |

**Figure S1. Funnel plot of mRS 0-2 at 90 days**

**Figure S2. Funnel plot of** **TICI 2b-3**

**Figure S3. Funnel plot of** **90-day mortality**

**Figure S4. Funnel plot of** **sICH**

**Figure S5. Forest plot of prospective cohort studies versus retrospective cohort studies for two treatment modalities.**

**Figure S6. Sensitivity analysis of revascularization rate**
